# Supplementary material for: Inferring strategies from observations in long iterated Prisoner’s dilemma experiments
Source: Sci Rep. 2022 May 9;12:7589. doi: 10.1038/s41598-022-11654-2 (PMC9085774; doi:10.1038/s41598-022-11654-2)
Supplement: Supplementary file 1 — Supplementary Information. [file 41598_2022_11654_MOESM1_ESM.pdf]

Supplementary Information: Inferring Strategies from Observations in Long Iterated Prisoner's Dilemma Experiments

Eladio Montero-Porras[1,*], Jelena Grujić[1,*], Elias Fernandez-Domingos[1,2], and Tom Lenaerts[1,2,3,4]

[1]Artificial Intelligence Laboratory, Vrije Universiteit Brussel, Brussels, 1050, Belgium
[2]Machine Learning Group, Université Libre de Bruxelles, Brussels, 1050, Belgium
[3]Center for Human-Compatible AI, UC Berkeley, Berkeley, 94702, USA
[4]FARI Institute, Université Libre de Bruxelles-Vrije Universiteit Brussel, 1050 Brussels, Belgium

March 21, 2022

Contents

1 Methods **2**
1.1 Experimental data . . . 2
1.2 Experimental data sessions and description. . . . 3
1.3 Modularity Network . . . 3
1.4 Algorithm for the Hidden Markov Model per sub-cluster. . . . 4

2 Supplementary Results **5**
2.1 Clustering curves and dendograms . . . 5
2.2 TSNE and network plots per treatment . . . 7
2.3 Analysis of the actions in the first round per sub-cluster. . . . 10
2.4 Cumulative sum of payoff per treatment. . . . 11
2.5 Memory-one strategies per round window in Fixed Partners treatment . . . 12
2.6 Memory-one strategies per round window in Shuffled Partners treatment . . . 13

1 Methods

1.1 Experimental data

The data from these experiments were collected in Brussels, Belgium, at the Brussels Experimental Economics Laboratory (BEEL), part of the Vrije Universiteit Brussel (VUB). Twelve sessions were held (see Table S2 for a breakdown per session), in total 188 participants were recruited. The group of participants consisted of 48% female and 52% male. The average age in all sessions was 22±8 years old. The participants played the IPD on individual isolated laptops, designated to avoid any type of communication or arrangements by the participants, who remained anonymous throughout the experiment. At the end of the experiments, subjects were asked optional questions about their age, gender, and information about the game they just played (see the attached documents "Instructions of the experiment" for the detailed questions and answers, and "UserInformation" for details about the participants.).

The FP treatment consisted of 6 sessions where 92 participants played pairwise IPD with the same opponent. The SP treatment consisted of 6 sessions where 96 participants played the same IPD as FP but with a different opponent each round, hence the "shuffled" name. In the IPD one normally has a probability that the game is continued given by a parameter ω, producing an average of $(1-\omega)^{-1}$ rounds. Also, in our design, participants were not told exactly when the IPD would end, and we designed them in such a way that there were 100 rounds. Table S1 shows the payoff matrix containing the per round rewards used in the two treatments. For both treatments, participants could observe the actions of their partner in the previous round, even when this partner changed from the previous to the current round.

	**C**	**D**
**C**	3,3	0,4
**D**	4,0	1,1

Supplementary Table S1: Payoff matrix for both IPD treatments.

At the beginning of the experiment, every participant read a detailed instruction document with a small test at the end to make sure they understood the game dynamics. At the end of the experiment, participants were given their gains (mean = 7.85 euros, SD = 1.67 in the SP treatment and mean = 10.61, SD = 3.87 in the FP treatment) and a show-up fee of 2.5 euros. Most players spent a total of one hour from the moment they entered the computer room to the moment they were paid for their participation.

1.2 Experimental data sessions and description.

Treatment	Session	Number of participants
Fixed Partners	1	8
	2	16
	3	18
	4	14
	5	18
	6	18
**Total**		**92**
Shuffled Partners	1	10
	2	18
	3	16
	4	18
	5	16
	6	18
**Total**		**96**

Supplementary Table S2: Number of participants per experimental session

Treatment	Context previous round	Frequency	Cooperation	(%)
Fixed Partners	CC	4,122	3,910	94.86%
	CD	980	562	57.34%
	DC	980	320	32.27%
	DD	3,026	316	10.44%
Shuffled Partners	CC	806	647	80.27%
	CD	1,910	1,127	59.0%
	DC	1,910	290	15.18%
	DD	4,878	626	12.83%

Supplementary Table S3: Frequency table for each context in the previous round and the percentage of cooperation given the context, per treatment.

1.3 Modularity Network

As mentioned in the Methods section of the main paper, we performed different clustering methods to test the separation between participants and their behavior. In this case, we also used the Modularity Network (MN) clustering, especifically the Louvain method [1]. In our case, a network was built, where each node represented a subject on each treatment (FP and SP) and they were connected by a weighted edge. We used eight variables representing the context and the cooperation under each context used in the other clustering methods (K-Means and Hierarchical Clustering), represented by a vector S:

$$S = ((CC),(CD),(DC),(DD),(CC)C,(CD)C,(DC)C,(DD)C) \tag{1}$$

This way, the distance between participant i and participant j is: $d = ||S_i - S_j||$. The weight between the nodes is given by the inverse of this distance plus one, to avoid divisions by zero:

$$w = \frac{1}{d+1} \tag{2}$$

In MN clustering, one aims to maximize the modularity M, in this case, our clustering resulted in $M = 0.40$ for all treatments. To test the robustness of the clustering a randomized network was built over 100 iterations and the same process of getting the modularity was done, this random network resulted in $M = 0.264 \pm 0.006$. Figure S4 shows the overlap between the K-Means and the MN clustering.

1.4 Algorithm for the Hidden Markov Model per sub-cluster.

Algorithm 1 Hidden Markov Model per sub-cluster. Up to 4 hidden states were tested, and the selected number of states h corresponded to the number of different hidden states predicted by the model itself, a low number of hidden states is preferred, both to avoid overfitting and to facilitate interpretation. One last reason to pick fewer hidden states is that some of these states and their transition probabilities are small, which between runs can tend to zero, and it will end up leaving the hidden state disconnected. To ensure connectedness, no model was accepted with a probability of transition less or equal to 0.01. These models were fitted over one million trials and picked the one that resulted with the highest log-likelihood to fit the sequences.

```
for each sub-cluster s ∈ cluster c do
    X_train, X_test = data[sub-cluster == s]
    ll_max = 0, best_model = null
    for each h ∈ [4,3,2,1]                                  ▷ Number of Hidden States do
        model = MultinomialHMM(h)
        trans_matrix, emission_matrix, initial_probs = model.fit(X_train)
        ll = log_likelihood(model.predict(X_test))
        if ll > ll_max AND p ∈ trans_matrix >= 0.01: best_model = model
    end for
end for
```

2 Supplementary Results

2.1 Clustering curves and dendograms

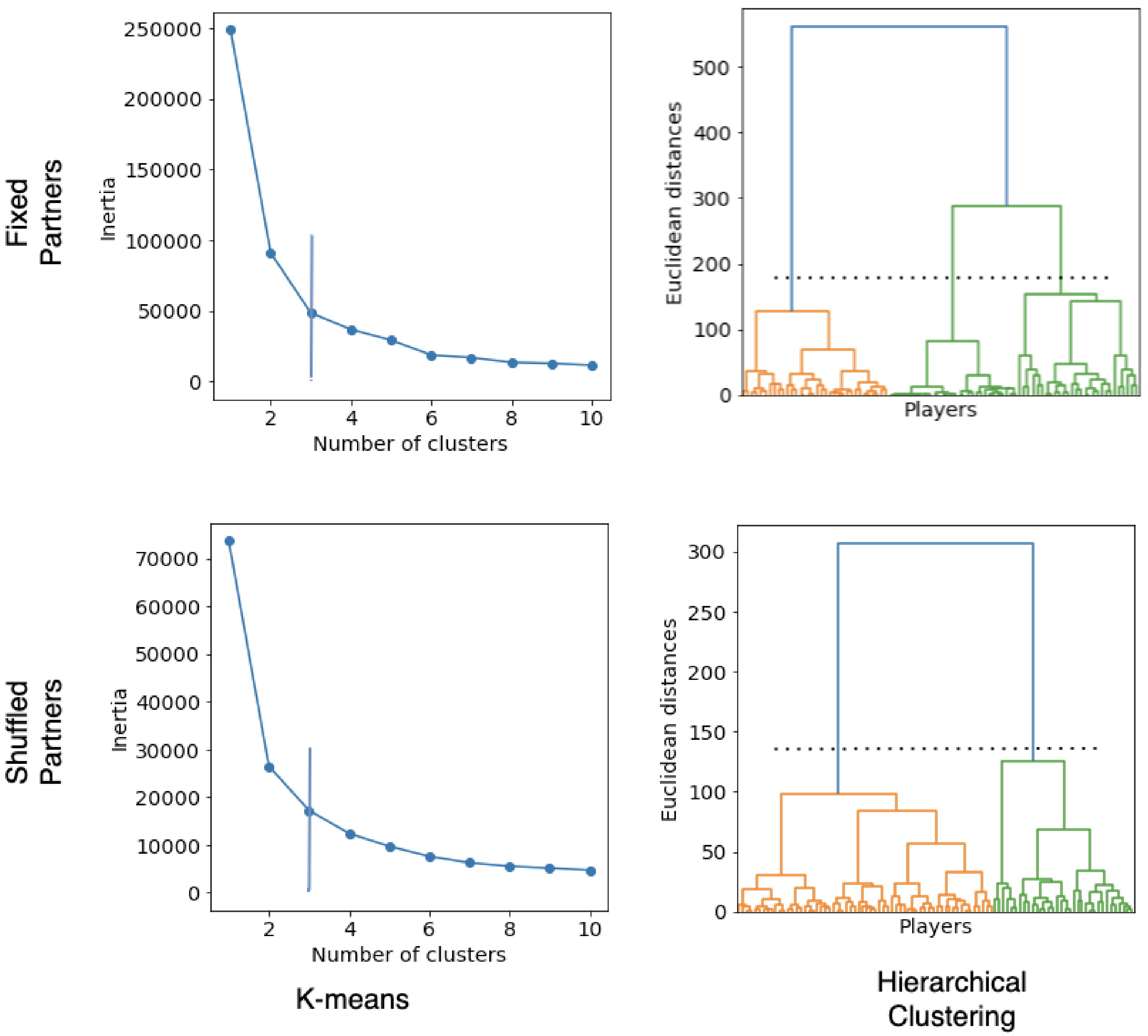

Supplementary Figure S1: Number of clusters per treatment. To test the number of clusters, in K-means (left panel) uses the ”elbow” method, where a range of different k (x-axis) are tested for their sum of squared distance between each data-point to its centroid, this is what is called inertia (y-axis). The vertical line represents the ”elbow” of the curve, as explained in the Methods section, or the point where the curve starts to stabilize. We used the Python library kneedle by Satopaa et al. [2] to find this point. Hierarchical clustering (right panel) uses a dendrogram, where all data points are plotted according to their distance to each other. To pick the number of clusters, one follows the largest vertical distance from the top and crosses an horizontal line (dotted line). Fewer clusters were preferred at this stage, for example picking 5 clusters in FP resulted in 0.5571 and picking 3 yielded 0.5531. For both K-means and Hierarchical Clustering, we used the scikit library for Python [3].

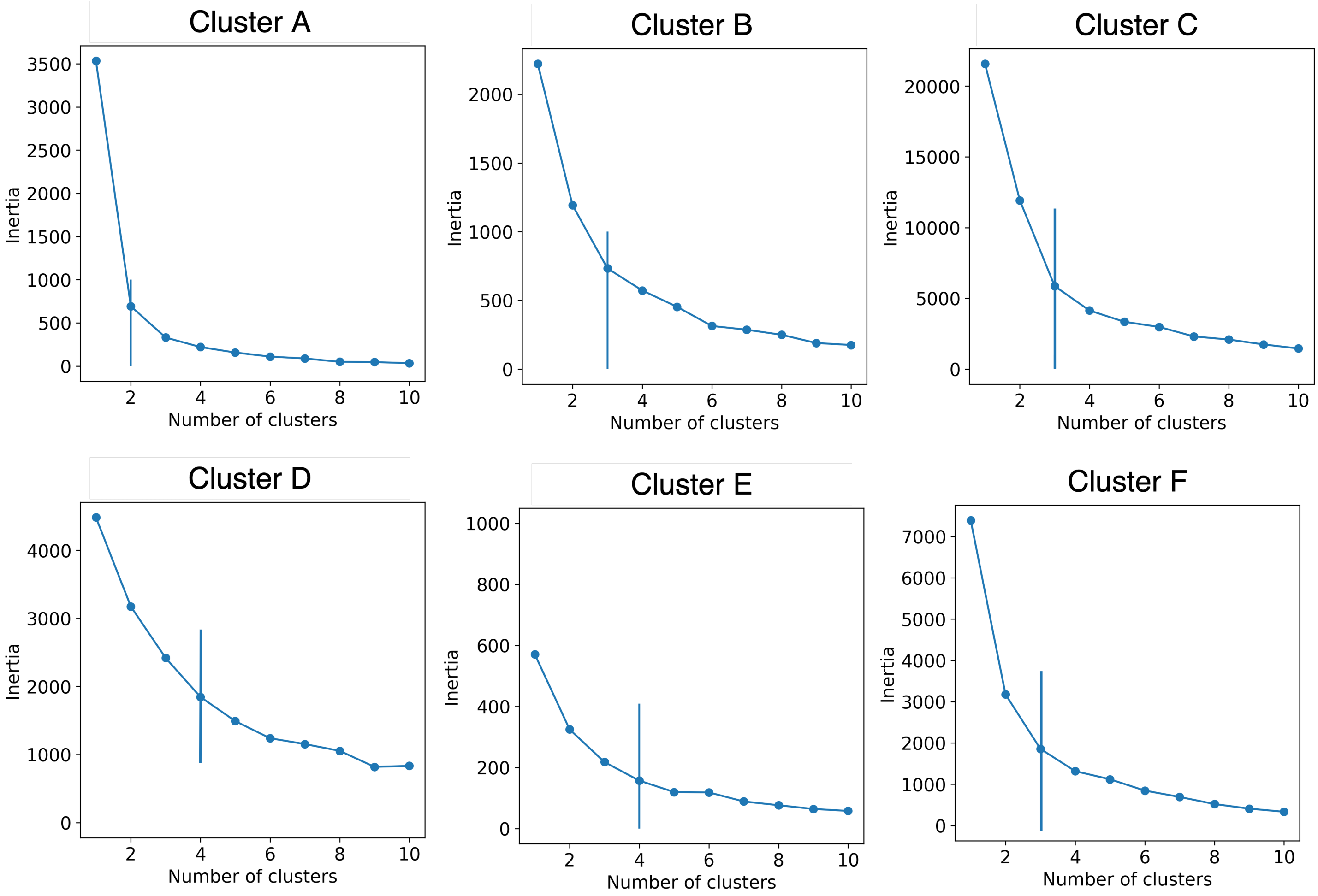

Supplementary Figure S2: Number of sub-clusters per cluster. A curve of inertia is plotted per cluster and per treatment. Inertia is the sum of squared distances of each data-point to their corresponding centroid in K-means (y-axis), and a range of different k are tested (x-axis). The vertical line represents the ”elbow” of the curve, as explained in the Methods section, or the point where the curve starts to stabilize. We used the Python library kneedle by Satopaa et al. [2] to find this point. In the top row, the FP is shown with its clusters: A, B, C; and in the bottom row the SP treatment with its clusters: D, E and F.

2.2 TSNE and network plots per treatment

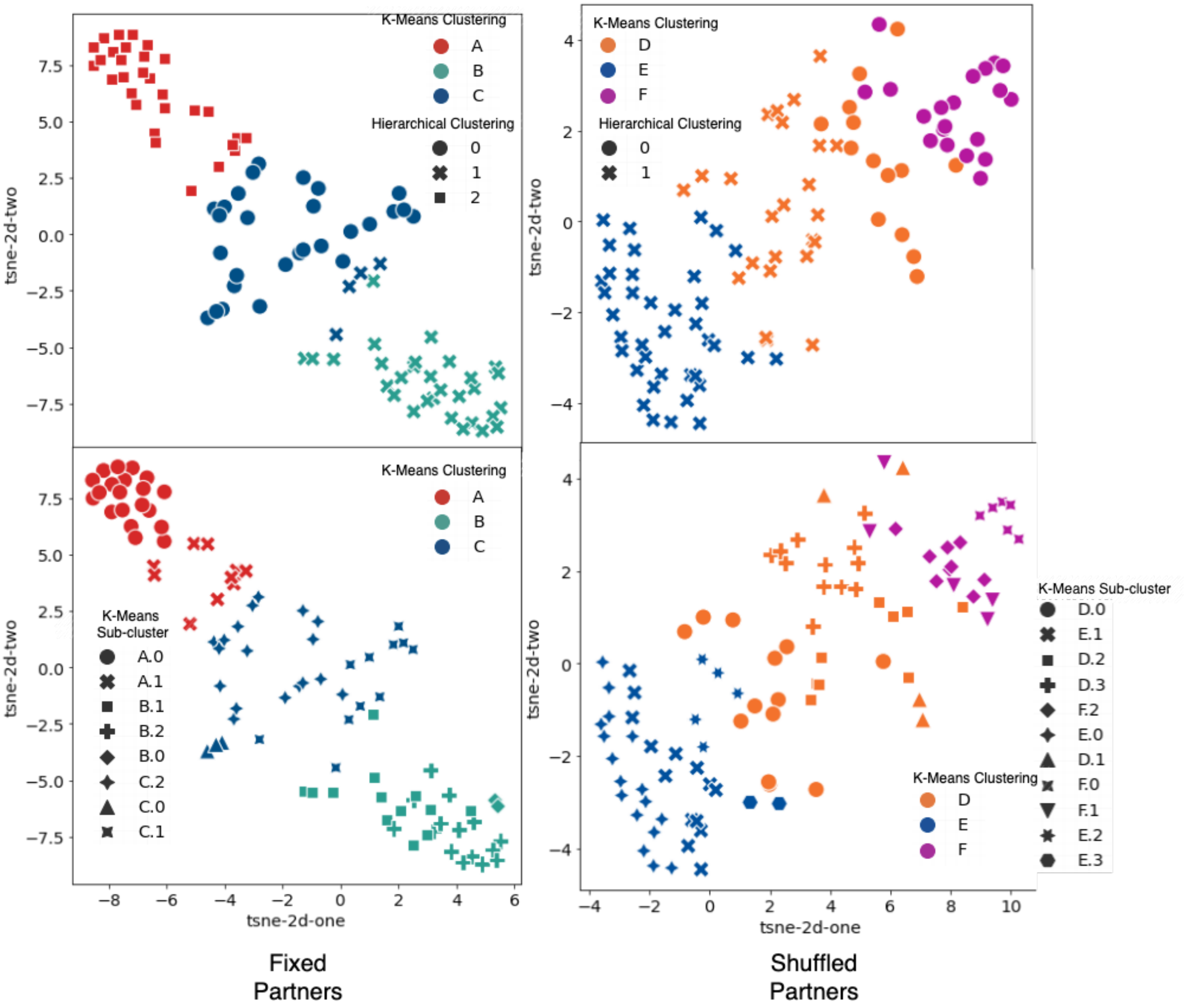

Supplementary Figure S3: tSNE components of both treatments reduced to two components. The tSNE plot was made with the context variables (CC, CD, DC and DD) and the cooperation variables (CCC, CDC, DCC, DDC), i.e. the number of times each subject faced each one of these scenarios in the previous round, for example, CD means that the focal player cooperated and their opponent defected in the previous round. In this case, these eight variables were reduced to two main components for visualization, each point represents a subject. In the top row, each color represents the classification in the K-Means clustering and its shape the hierarchical clustering method for comparison. The left panel in the top row shows the subjects of the FP treatment (clusters A, B and C of K-Means) and the right panel the SP treatment (clusters D, E and F of K-Means). The third row shows the behavioral clusterings. It is visible how the clustering made with different methods overlap in both treatments. In the row below, it is shown how the clusters in k-means are subdivided by their corresponding sub-clusters.

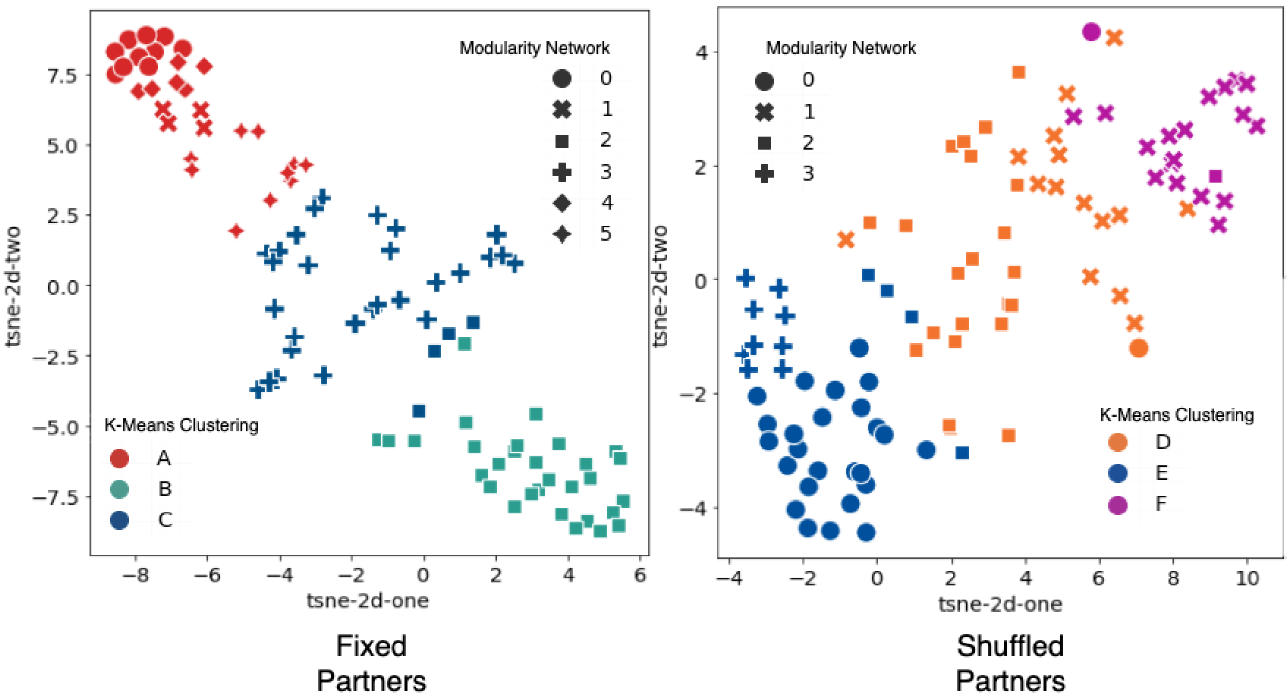

Supplementary Figure S4: tSNE Plot Modularity Network (MN) and K-Means clustering. As seen in the previous figure, the colors are the clusters found with K-Means and the shapes the clusters found with MN clustering. The overlap is still visible, even though they are two different approaches to unsupervised learning.

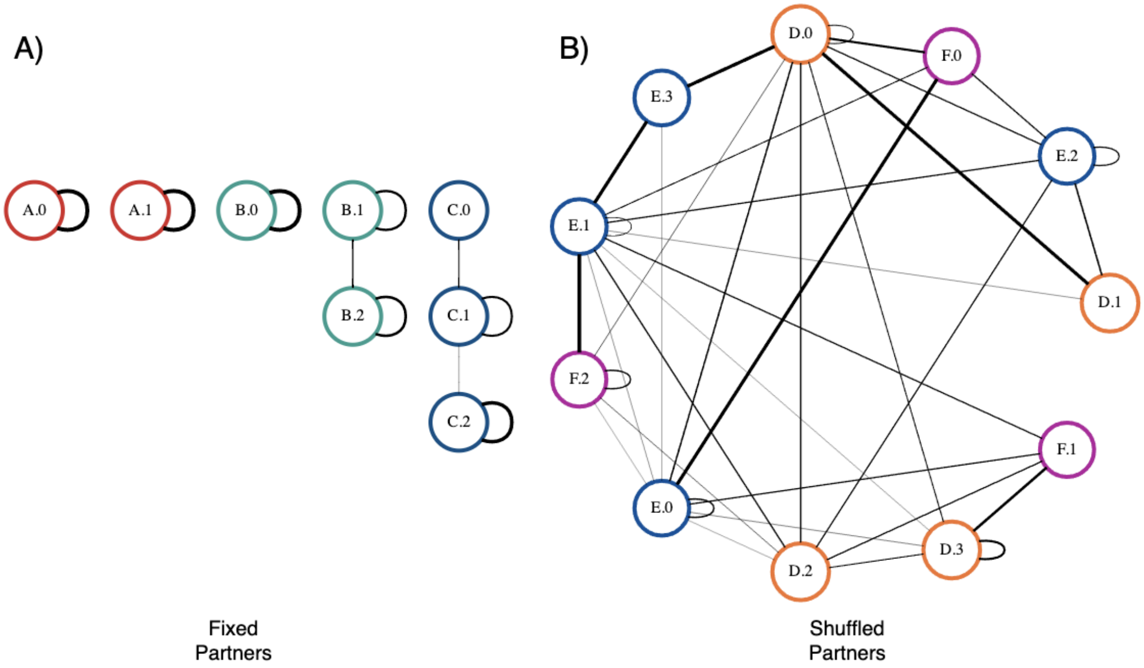

Supplementary Figure S5: Network showing the interaction between sub-clusters in both treatments. **A)** FP treatment and **B)** SP treatment. In SP, it can be seen how subclusters interact with members of other clusters, in contrast, a self-organisation can be seen in FP since members of the same sub-cluster interact with each other, or in the case of sub-clusters B.1, B.2, C.0, C.1 and C.2 interact with members of the same cluster (B and C respectively).

2.3 Analysis of the actions in the first round per sub-cluster.

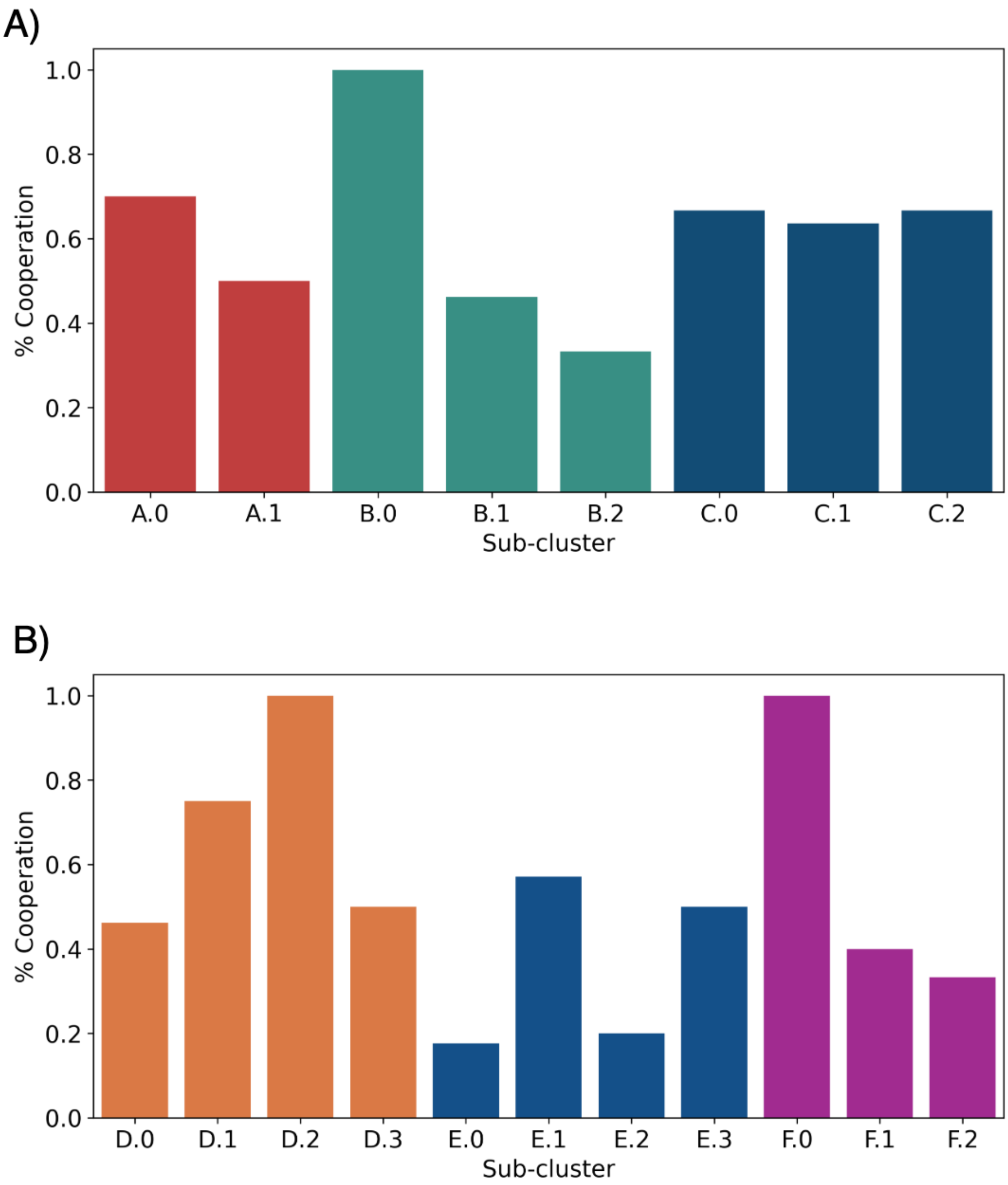

Supplementary Figure S6: First action per cluster and sub-cluster. A) Fixed partners and B) Shuffled Partners.

2.4 Cumulative sum of payoff per treatment.

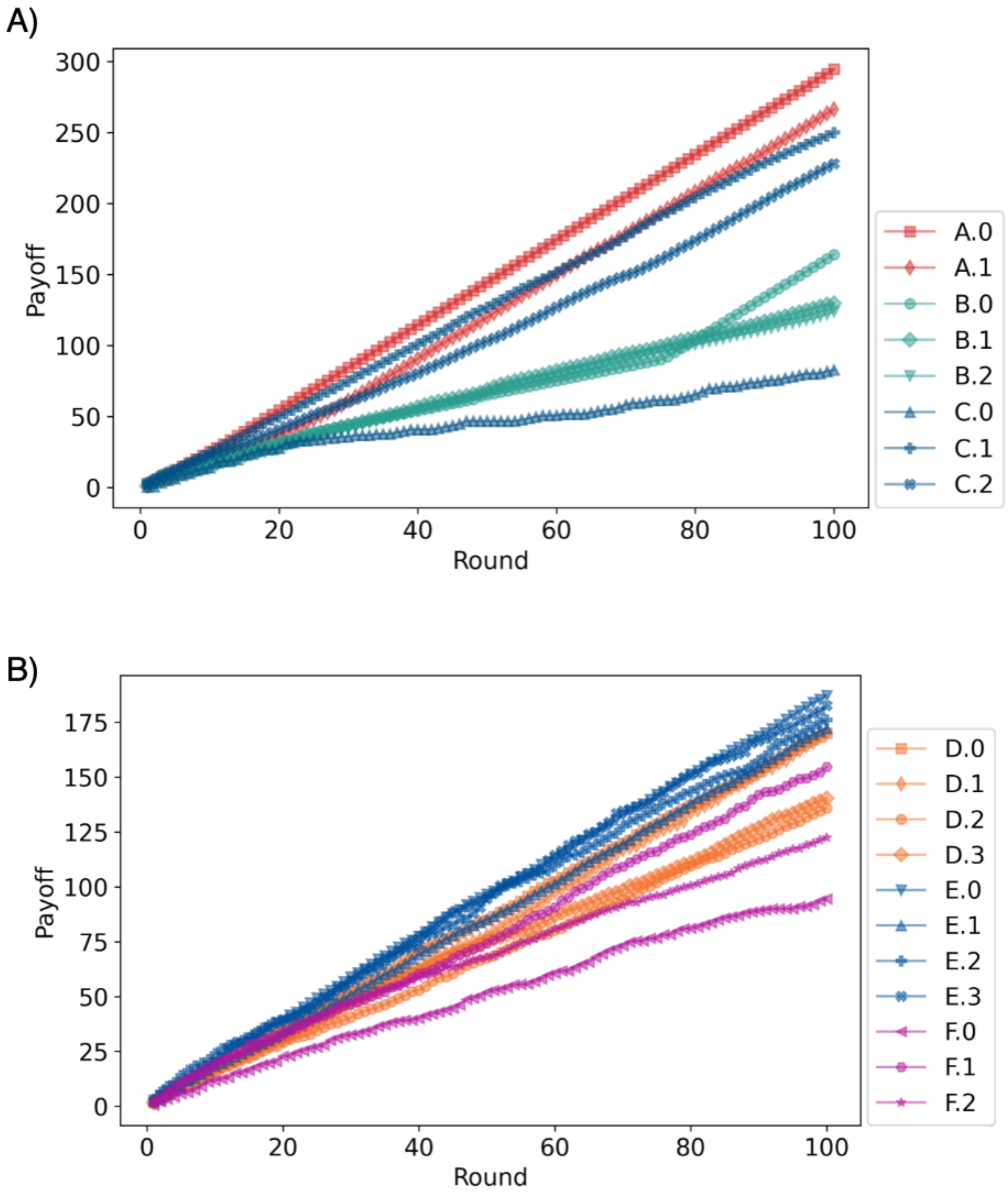

Supplementary Figure S7: Cumulative sum of payoff per treatment. A) Fixed partners and B) Shuffled Partners. We tested the hypothesis that subjects could change their behavior depending on how good or bad they were doing during the game. We found that for example, highly cooperative clusters such as A.0 and A.1 found themselves in a diagonal line, meaning that their earnings were stable during the game. While in cluster such as C or F in SP, depending on the sub-cluster a steeper (or flatter) line was drawn.

2.5 Memory-one strategies per round window in Fixed Partners treatment

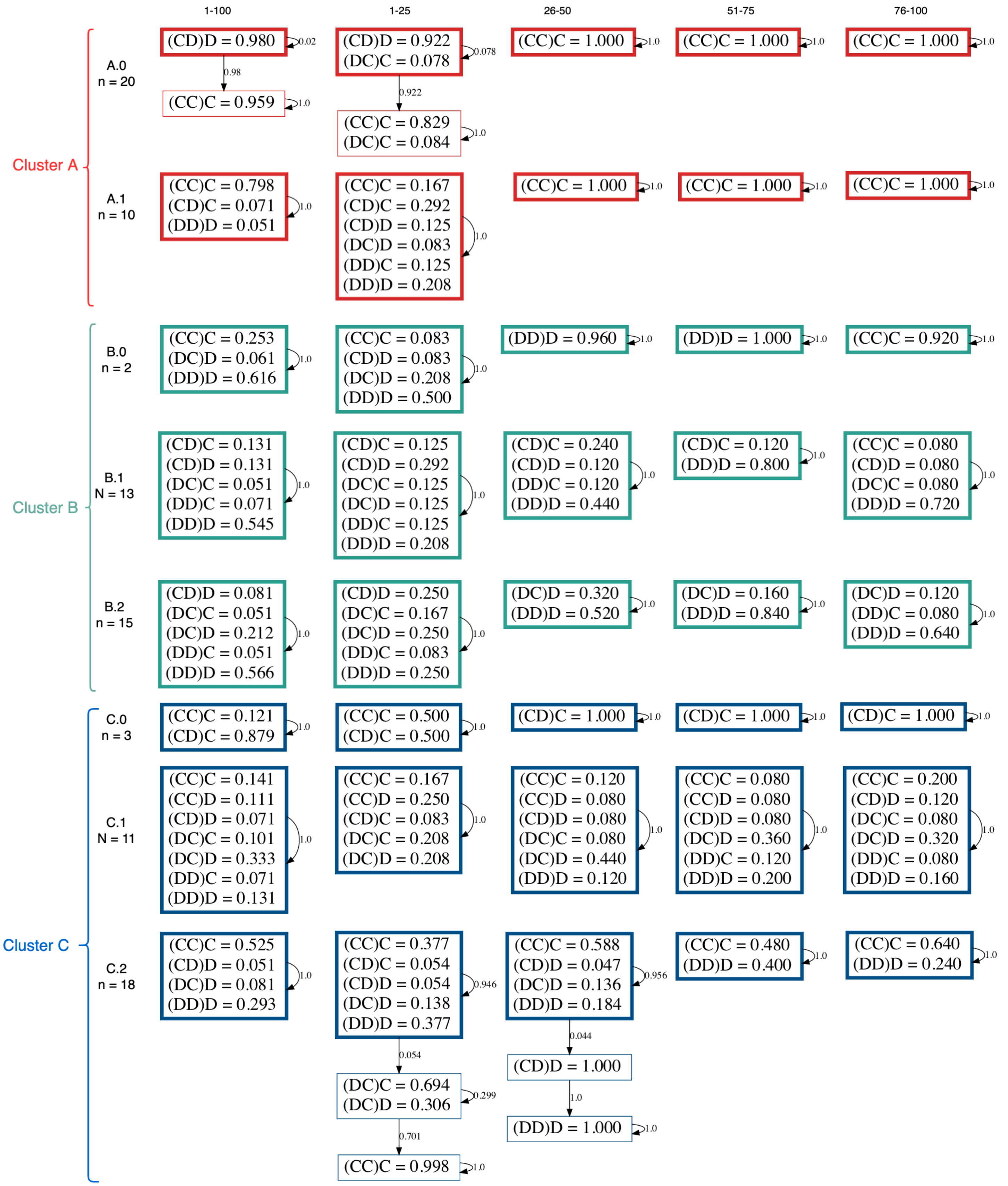

Supplementary Figure S8: Hidden Markov Models for the Fixed Partners treatment. Here, the eight sub-clusters found in FP have a HMM that describes each sub-cluster's strategy. Bold rectangles represent the initial state, while the others represent subsequent hidden states. At the top, round windows are specified, starting from 1-100, then in windows of 25.

2.6 Memory-one strategies per round window in Shuffled Partners treatment

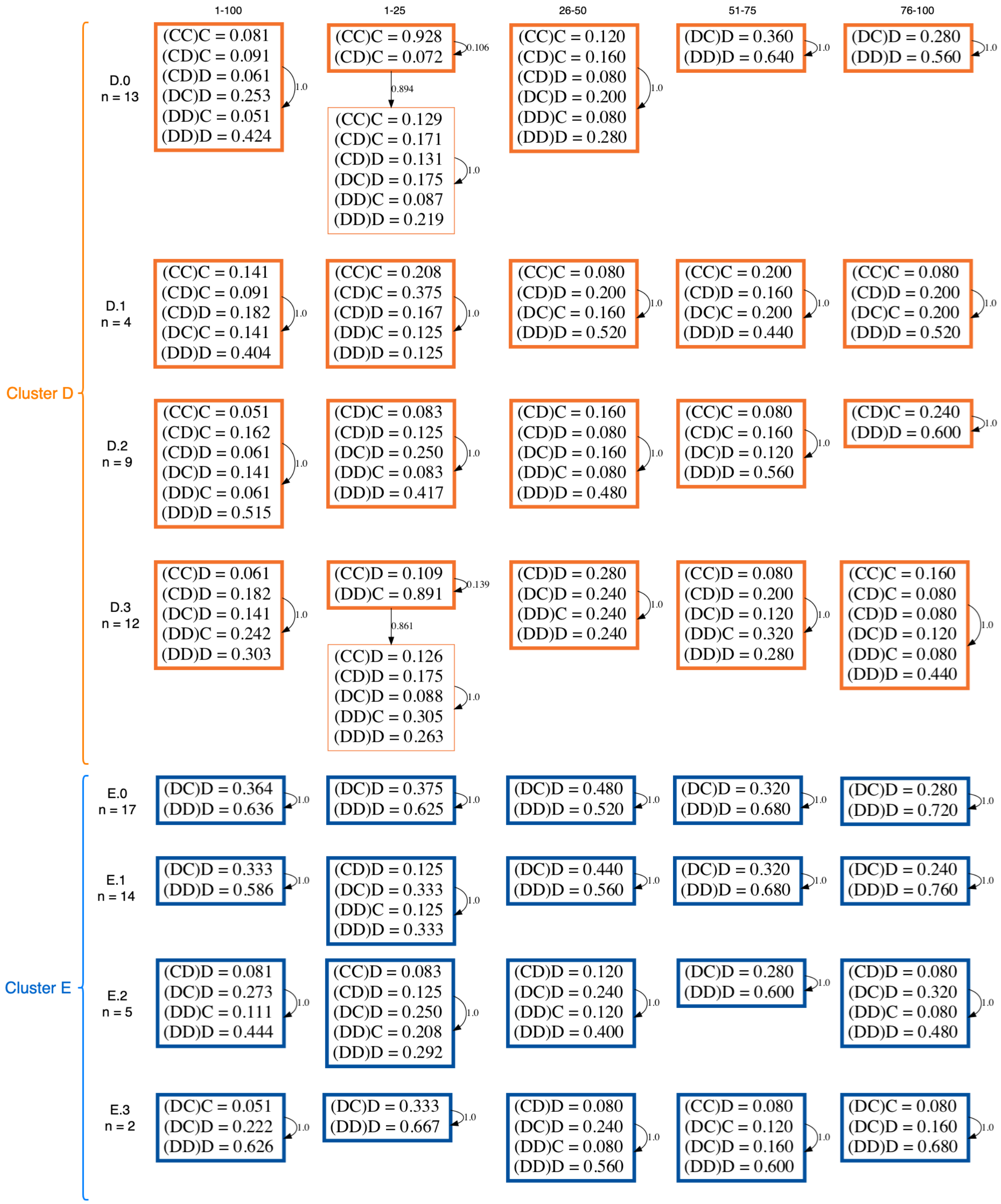

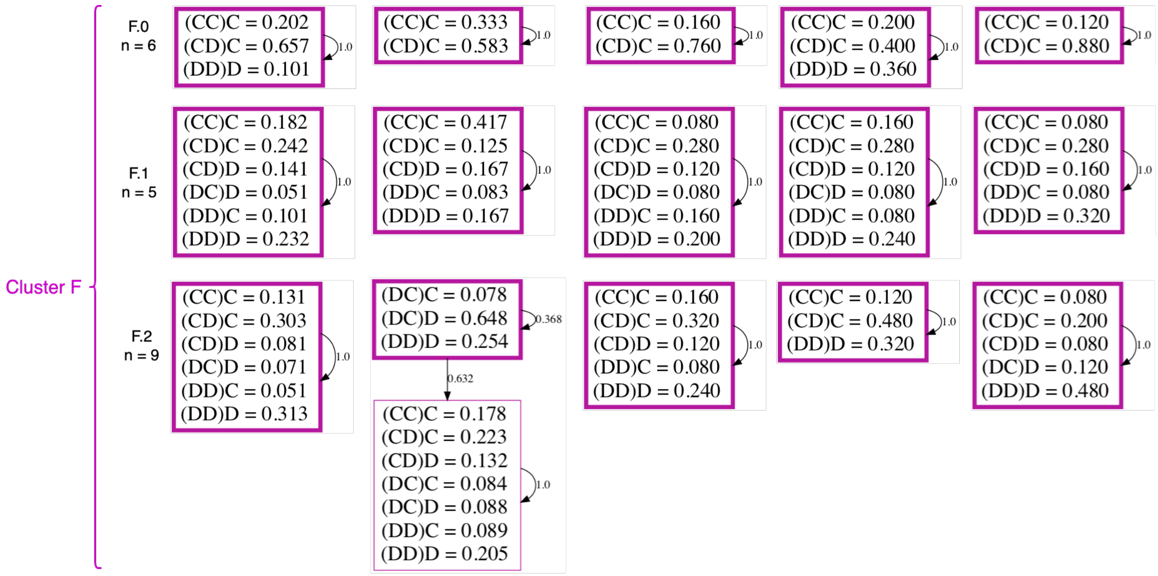

Supplementary Figure S9: Hidden Markov Models for the Shuffled Partners treatment. Here, the ten sub-clusters found in SP have a HMM that describes each sub-cluster's strategy. Bold rectangles represent the initial state, while the others represent subsequent hidden states. At the top, round windows are specified, starting from 1-100, then in windows of 25.

References

1. Blondel, V. D., Guillaume, J.-L., Lambiotte, R. & Lefebvre, E. Fast unfolding of communities in large networks. en. *Journal of Statistical Mechanics: Theory and Experiment* **2008.** Publisher: IOP Publishing, P10008. ISSN: 1742-5468. https://doi.org/10.1088/1742-5468/2008/10/p10008 (2021) (Oct. 2008).
2. Satopaa, V., Albrecht, J., Irwin, D. & Raghavan, B. *Finding a "Kneedle" in a Haystack: Detecting Knee Points in System Behavior* in *2011 31st International Conference on Distributed Computing Systems Workshops* ISSN: 2332-5666 (June 2011), 166–171.
3. Pedregosa, F. *et al.* Scikit-learn: Machine Learning in Python. *The Journal of Machine Learning Research* **12,** 2825–2830. ISSN: 1532-4435. https://scikit-learn.org/stable/ (Nov. 2011).
